# Development of Ammonia Emission Factor for Industrial Waste Incineration Facilities Considering Incinerator Type

**DOI:** 10.3390/ijerph19105949

**Published:** 2022-05-13

**Authors:** Joonyoung Roh, Seongmin Kang, Buju Gong, Kyungwon Lee, Eui-chan Jeon

**Affiliations:** 1 Department of Climate and Environment, Sejong University, Seoul 05006, Korea; shdod88@naver.com; 2 Climate Change & Environment Research Center, Sejong University, Seoul 05006, Korea; smkang9804@gmail.com; 3 Air Quality Research Division, National Institute of Environmental Research, Incheon 22689, Korea; bjkong@korea.kr (B.G.); ruds83@korea.kr (K.L.)

**Keywords:** PM_2.5_ secondary sources, industrial waste incinerator, NH_3_ emission factor

## Abstract

In this study, the emission factor and concentration of ammonia from industrial waste incineration facilities were analyzed through actual measurements. The ammonia emission factor was calculated and the difference in ammonia emission factor for each type of incineration was confirmed through the Mann–Whitney U test. As a result of analyzing 279 samples, the NH_3_ emission factor of the SNCR facility of stoker types was 0.012 kgNH_3_/ton, and the NH_3_ emission factor of the SNCR facility of the rotary kiln methods was 0.014 kgNH_3_/ton. Additionally, the NH_3_ emission factor of this study was higher than the NH_3_ emission factor (0.003 kgNH_3_/ton) suggested by Kang’s study (0.009 kgNH_3_/ton) and EMEP/EEA (2006). There is a need to develop an NH_3_ emission factor that takes into account the characteristics of Korea, since it is largely different from the NH_3_ emission factor of EMEP/EEA. As a result of statistical analysis of the stoker type and the rotary kiln method, the null hypothesis that there is no difference between each type was adopted (*p*-value > 0.05), indicating that there was no statistical difference in the ammonia emission factors of the stoker type and the rotary kiln type.

## 1. Introduction

According to the 2019 World Air Quality Report published by IQAir in 2019, 61 cities in South Korea with high concentrations of fine dust (PM_2.5_) were included in the top 100 cities affiliated to OECD member countries. Compared to 2018, 17 cities were added to the list, indicating that air pollution has not been alleviated [1].

Fine dust (PM_2.5_) can be divided into direct emissions from emission sources and indirect emissions (secondary production) from chemical reactions involving NH_3_, sulfur oxides, nitrogen oxides, and volatile organic compounds. Indirect emissions account for approximately 72% of the total fine-dust emissions, and the largest emissions result from NH_3_ reacting with sulfur and nitrogen oxides [2,3,4].

Recently, many management policies have been implemented due to the increase in PM_2.5_ concentration, and in the case of Korea, various efforts are being made, such as preparing many measures to reduce PM_2.5_ and establishing related laws.

In relation to the above, although sulfur and nitrogen oxides are relatively well-managed, NH_3_ is controlled as a substance generating odor and air pollution, implying that the allowable emission concentrations are high and there is insufficient related emission control and research. Therefore, it is necessary to conduct research on NH_3_ emission calculation and the development of emission factors [5,6,7].

Waste disposal methods include incineration, landfill, and recycling. Selective catalytic reduction (SCR) and selective non-catalytic reduction (SNCR) techniques are used to remove nitrogen oxides, which are air pollutants emitted during incineration processes. If an excess amount of NH_3_ is used during the process, it is emitted into the atmosphere [8,9,10,11].

However, NH_3_ emissions are not considered when calculating the total amount of air pollutants emitted through industrial waste incineration in Korea [12]. Furthermore, as SCR and SNCR equipment are used in industrial waste incineration facilities, the development of related emission factors and emission calculations is necessary. Although studies on ammonia emission factors of MSW or sludge incineration facilities have been conducted, it has been found that no studies have been conducted on ammonia emission factors of industrial waste incineration facilities [13,14].

Therefore, this study attempted to analyze the emission concentration and develop the NH_3_ emission factors in industrial waste incineration facilities, and to use statistical methods to calculate NH_3_ emission factors and determine the difference in NH_3_ emission factor for each type of incinerator.

## 2. Method

### 2.1. Selection of Objective Facilities

NH_3_ emission factors by incinerator type in industrial waste incineration facilities were calculated by acquiring process data such as concentration, TMS (Tele-Monitoring System) data, and fuel usage. The incinerator types were classified as presented in Table 1. We selected the stoker and rotary kiln incinerators. A total of 179 samples were analyzed, of which 136 and 43 samples were obtained from the stoker and rotary kiln incinerators, respectively. Ammonia concentration measurement data are for 3 years (2017~2019).

### 2.2. NH_3_ Analysis in Industrial Waste Incinerators

In this study, the indophenol method, which is official method in Korea, was used to analyze the concentration of NH_3_ [15,16]. The amount of NH_3_ was calculated by adding sodium hypochlorite and phenol-sodium nitroprusside solutions to the sample solution, and by measuring the absorbance of the indophenols produced by the reaction with NH_3_ ions. The NH_3_ samples were collected by placing NH_3_ absorption liquids (a standard boric acid solution of 50 mL that can absorb) into two volumetric flasks and using a mini pump (SIBATA MP-ΣNII, Saitama, Japan) to suck 50 L of exhaust gas for 20 min at a rate of 2.5 L/min.

**Figure 1:**
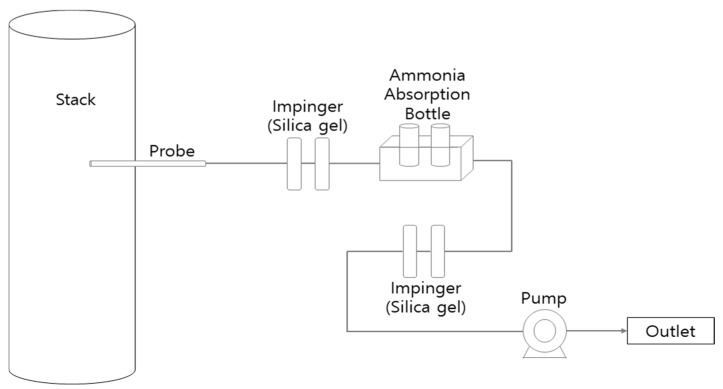
Schematic of the field setup for ammonia sampling at waste incinerator.

A bottle filled with silica gel was installed in front of the collected NH_3_ sample to remove moisture from the samples [17]. A schematic diagram on acquiring NH_3_ samples is illustrated in Figure 1. The NH_3_ concentration was determined by measuring the absorption in the absorption liquid using a spectrophotometer (Shimadzu 17A, Kyoto, Japan) with a wavelength of 640 nm.

### 2.3. Development of NH_3_ Emission Factor

Mathematical formulae used in studies related to the development of NH_3_ emission factors were referred to, and Equation (1) was used to calculate the NH_3_ emission factors [18,19]. The NH_3_ concentration, flow rate, and amount of waste incinerated are required to calculate the NH_3_ emission factors of industrial waste incineration facilities.

CleanSYS, which is operated to control air pollutants in Korea, measures the concentrations of sulfur oxides, particulate matter, and nitrogen oxides, and the flow rate and temperature of the exhaust gas in real-time [20]. The cumulative flow rate data for a single day were used with reference to CleanSYS. In this study, the indophenol method was used to measure the NH_3_ concentration because CleanSYS does not currently measure the NH_3_ concentration. The data on the amount of industrial waste were obtained through the target business site.

(1) $$EF_{{\mathrm{NH}}_{3}}=\left[C_{{\mathrm{NH}}_{3}}\times \frac{M_{w}}{V_{m}}\times Q_{day}\times 10^{-6}\right]/FC_{day}\;$$

where EF is emission factor (kg NH_3_/ton); $C_{{\mathrm{NH}}_{3}}$ is NH_3_ concentration in flue gas (ppm); $M_{w}$ is molecular weight of NH_3_ (constant) = 17.031 (g/mol); $V_{m}$ is one mole ideal gas volume in standardized condition (constant) = 22.4 (10^−3^ m^3^/mol); $Q_{day}$ is daily accumulated flow rate (Sm^3^/day) (based on dry combustion gas); and $FC_{day}$ is a daily amount of industrial waste (ton/day).

### 2.4. Statistical Analysis Method for Incinerator Type

**Figure 2:**
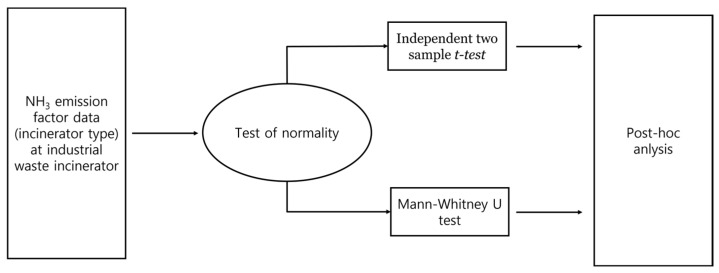
Schematic of statistics analysis.

The average distributions of the NH_3_ emission factor for each type of incinerator were compared to investigate whether the incinerator type of the industrial waste incineration facilities affects the NH_3_ emission factor. SPSS 21 was used to compare the average distributions, and the statistical procedures for comparing the average distribution of the NH_3_ emission factor by incinerator type are shown in Figure 2 [14]. In this study, after testing the normality of the ammonia emission concentration data, an average comparison analysis method was used that fits the normality result.

## 3. Results and Discussion

### 3.1. NH_3_ Emission Factors of Industrial Waste Incineration Facilities

In this study, the NH_3_ emission factors were calculated using the NH_3_ concentration and the data obtained from industrial waste incineration facilities, and the results are presented in Table 2. NH_3_ emission factors of the industrial waste incineration facilities were 0.012 and 0.014 kgNH_3_/ton for the stoker and rotary kiln incinerators, respectively.

Currently, there are no comparable data because the NH_3_ emission factor for industrial waste incinerators is not calculated in Korea. Therefore, the NH_3_ emission factor of the incineration of municipal solid waste calculated in a related study was used for comparison with the NH_3_ emission factor listed in the EMEP/EEA (2006) of Europe [21]. As presented in Table 3, the NH_3_ emission factors obtained in this study were observed to be higher than the values obtained by Kang et al., and the value listed in the EMEP/EEA (2006) [18,19,21].

### 3.2. Normality Test for NH_3_ Emission Factors of Industrial Waste Incineration Facilities

The normality of the data must be tested for statistical analysis of the calculated NH_3_ emission factors. The K-S test is typically used if there are more than 2000 datapoints, whereas the Shapiro–Wilk test is used if there are less than 2000 [22,23].

The normality of the data can be determined by assuming the null hypothesis that states the population is normally distributed. If significance is >0.05, a normal distribution is assumed; however, if significance is <0.05, the null hypothesis is rejected, and the population distribution is considered non-normal.

A statistical program (SPSS 21) was used in this study, and the Shapiro–Wilk method was used to determine the normality because the number of samples for each type of waste incinerator was less than 2000 [22,23]. Normality test results showed that the values obtained for both the stoker and rotary kiln incinerators used in the incineration of industrial waste had a significance of less than 0.05, indicating that they do not fol-low a normal distribution, and the results are presented in Table 4.

### 3.3. Mann–Whitney U Test of NH_3_ Emission Factor by Incinerator Type

The normality test results of the NH_3_ emission factors by incinerator type showed that all distributions were non-normal. Therefore, the difference between the two groups was determined using the Mann–Whitney U test, typically used when normality is not met, and the results are presented in Table 5.

The analysis results showed that significance was >0.05, indicating that the null hypothesis that states “there is no difference in NH_3_ emission factors between the two incinerator types” was accepted. Thus, there is no difference in NH_3_ emission factors between the stoker and rotary kiln types.

## 4. Conclusions

In this study, NH_3_ emission factors were calculated for two types of incinerator used in industrial waste incineration facilities in Korea, as NH_3_ emission factors are currently not applied, and the statistical difference between the two emission factors obtained was analyzed.

Based on the classification of industrial waste incinerators, the NH_3_ emission factors were calculated for the stoker and rotary kiln incinerators, and all facilities were investigated using SNCR equipment. A total of 179 samples were acquired, of which 136 and 43 samples were from the stoker and rotary kiln incinerators, respectively:

1. The results showed that the NH_3_ emission factor of SNCR equipment in stoker incinerators was 0.012 kgNH_3_/ton, whereas that for the rotary kiln incinerators was 0.014 kgNH_3_/ton. Because the NH_3_ emission factor of the incineration of industrial waste is not currently applied in Korea, the NH_3_ emission factor of municipal solid waste incineration reported in another study was used for comparison with the NH_3_ emission factor listed in the EMEP/EEA (2006) of Europe.
2. Comparison of the results showed that the NH_3_ emission factors of this study were higher than those reported by Kang et al., (0.009 kgNH_3_/ton) and the value stated in the EMEP/EEA (0.003 kgNH_3_/ton) (2006). In particular, the NH_3_ emission factor obtained in this study was vastly different from the value listed in the EMEP/EEA, which was measured abroad, indicating that the development of NH_3_ emission factors needs to be conducted considering the characteristics of Korea.
3. The difference in NH_3_ emission factors between the stoker and rotary kiln incinerators was analyzed statistically. The Mann–Whitney U test was used for analysis, as values for both incinerator types showed a non-normal distribution. The results showed that the null hypothesis, stating there is no difference between the two types, was accepted (*p*-value > 0.05), indicating that there was no statistical difference between the NH_3_ emission factors of the stoker and rotary kiln incinerators.

Some of the industrial waste incineration facilities in Korea were found to use fluidized bed incinerators, but no data related to the fluidized bed incinerators were obtained in this study. Therefore, NH_3_ emission of fluidized bed incinerators should be addressed in future studies, followed by statistical analysis of the differences between NH_3_ emission factors among incinerator types and a determination of whether emission factors need to be developed for each type of incinerator. Furthermore, research on the calculation of NH_3_ emission factors in solid waste and sewage sludge incineration facilities is expected to enhance the reliability of the NH_3_ inventory in the field of waste management.

## Figures and Tables

**Table 1:** Sampling status of objective facilities.

Waste Type	Incinerator Type	De-Nox Facilities	Sampling
Industrial Waste	Stoker	SNCR	136
	Rotary Kiln	SNCR	43
Total			179

**Table 2:** NH_3_ emission factor by type of incineration at industrial waste incinerator.

Incinerator Type	NH_3_ Emission Factor	N (Unit: kgNH_3_/ton)
Stoker	0.012	136
Rotary Kiln	0.014	43

**Table 3:** Comparison of NH_3_ emission factors at waste incinerator.

Classification	Waste Type	Incinerator Type	NH_3_ Emission Factor (Unit: kgNH_3_/ton)
This study	Industrial Waste	Stoker	0.012
		Rotary Kiln	0.014
Kang et al. (2020)	MSW	Stoker	0.009
EMEP/EEA (2006)		-	0.003

**Table 4:** The result of normality test on NH_3_ emission factor data by incinerator type at industrial waste incinerators.

Normality Test Result	Shapiro–Wilk		
	Statistic	Degrees of Freedom, Df	Sig.
Stoker	0.948	136	0.000
Rotary Kiln	0.808	43	0.000

**Table 5:** The result of Mann–Whitney U test by NH_3_ emission factor by industrial waste incinerator type.

Incinerator Type		Mean ± SD	Z	*p*-Value
Stoker	SNCR	0.012 ± 0.006	−1.374	0.169
Rotary Kiln	SNCR	0.014 ± 0.016		

## Data Availability

Date sharing not applicable.
